# Autocrine Production of PDGF Stimulated by the Tenascin-C-Derived Peptide TNIIIA2 Induces Hyper-Proliferation in Glioblastoma Cells

**DOI:** 10.3390/ijms20133183

**Published:** 2019-06-28

**Authors:** Motomichi Fujita, Tetsuya Yamamoto, Takuya Iyoda, Tatsuya Fujisawa, Reo Nagai, Chikako Kudo, Manabu Sasada, Hiroaki Kodama, Fumio Fukai

**Affiliations:** 1 Department of Molecular Patho-Physiology, Faculty of Pharmaceutical Sciences, Tokyo University of Science, 2641 Yamazaki, Noda, Chiba 278-8510, Japan; 2 Department of Neurosurgery, Graduate School of Medicine, Yokohama City University, 3-9 Fukuura, Kanazawa-ku, Yokohama, Kanagawa 236-0004, Japan; 3 Department of Pharmacy, Faculty of Pharmaceutical Sciences, Sanyo-Onoda City University, 1-1-1 Daigaku-Doori, Sanyo-Onoda, Yamaguchi 756-0884, Japan; 4 Faculty of Science and Engineering, Saga University, 1 Honjo-machi, Saga-city, Saga 840-8502, Japan; 5 Translational Research Center, Research Institutes for Science and Technology, Tokyo University of Science, 2641 Yamazaki, Noda, Chiba 278-8510, Japan

**Keywords:** tenascin-C, β1-integrin, glioblastoma, PDGF, PDGF receptor

## Abstract

Expression level of tenascin-C is closely correlated to poor prognosis in glioblastoma patients, while the substantial role of tenascin-C responsible for aggressive progression in glioblastoma cells has not been clarified. We previously found that peptide TNIIIA2, which is derived from the tumor-associated tenascin-C variants, has the ability to promote cell adhesion by activating β1-integrins. Our recent study demonstrated that potentiated activation of integrin α5β1 by TNIIIA2 causes not only a dysregulated proliferation in a platelet-derived growth factor (PDGF)-dependent manner, but also disseminative migration in glioblastoma cells. Here, we show that TNIIIA2 enhances the proliferation in glioblastoma cells expressing PDGF-receptorβ, even without exogenous PDGF. Mechanistically, TNIIIA2 induced upregulated expression of PDGF, which in turn stimulated the expression of tenascin-C, a parental molecule of TNIIIA2. Moreover, in glioblastoma cells and rat brain-derived fibroblasts, tenascin-C upregulated matrix metalloproteinase-2, which has the potential to release TNIIIA2 from tenascin-C. Thus, it was shown that autocrine production of PDGF triggered by TNIIIA2 functions to continuously generate a functional amount of PDGF through a positive spiral loop, which might contribute to hyper-proliferation in glioblastoma cells. TNIIIA2 also enhanced *in vitro* disseminative migration of glioblastoma cells *via* the PKCα signaling. Collectively, the tenascin-C/TNIIIA2 could be a potential therapeutic target for glioblastoma.

## 1. Introduction

Tenascin-C, which belongs to the matricellular family of proteins, is poorly expressed in normal adult tissues but highly expressed in both tumor cells and tumor associated-stromal cells of many malignant tumors, such as glioma and breast and lung cancers, and its expression levels are associated with a poor prognosis in patients with these tumors [1,2,3,4]. Alternative splicing of fibronectin-type III (type III) repeats of tenascin-C generates a number of splice variants [5]. Among them, tenascin-C variants containing the alternatively spliced domain type III-A2 are highly expressed in malignant tumors [6,7]. Tenascin-C variant molecules containing the type III-A2 domain have therefore been implicated in tumorigenesis and tumor progression, while the substantial role of these variants in oncogenic transformation and malignant progression has remained elusive.

Glioblastoma multiforme (GBM) is the most common and aggressive primary malignant brain tumor in adults. Despite multimodality treatments, including surgery, irradiation, and chemotherapy, patients with GBM have a poor prognosis with a median survival time of less than 20 months [8,9,10]. Dysregulated proliferation and disseminative migration, which limit the potential for surgical resection, have been implicated in the devastating prognosis for GBM patients. There is an urgent need for new therapies in accordance with the identification of mechanisms involved in aggressive progression. The acquisition of highly aggressive behavior in GBM, such as dysregulated proliferation and disseminative migration, might be influenced by the tumor microenvironment, particularly the adhesive microenvironment affected by the extracellular matrix (ECM) expressed in tumor stroma. Because tenascin-C is highly expressed in GBM and its expression levels closely correlate with a poor prognosis in GBM patients [1], it has long been considered to be a negative prognostic factor in cancers including GBM. Nevertheless, the biological functions of tenascin-C in the aggressive progression of GBM have not been established.

We previously found that a peptide derived from the tumor-associated type III-A2 domain of the tenascin-C molecule, termed TNIIIA2, is capable of inducing conformational change in β1-integrins that are necessary for its functional activation [11,12]. Recently, we demonstrated that the activation of integrin α5β1 by TNIIIA2 induces hyper-stimulation of platelet-derived growth factor (PDGF)-dependent proliferation and disseminated migration in GBM cells, which are crucial determinants of a poor prognosis in GBM, thus suggesting that the TNIIIA2-induced activation of integrin α5β1 might cause aggressive progression in these cells [13]. In addition, PDGF and PDGF-receptor (PDGF-R) are highly expressed in GBM [14,15]. In some subgroups of GBM, PDGF signaling pathway has been reported to be dysregulated [16,17], therefore, the TNIIIA2/β1-integrin/PDGF-R axis might serve as a therapeutic target.

Here, we show that activation of β1-integrins by TNIIIA2 strongly enhances cell proliferation in PDGF-Rβ-expressing GBM cells, even without exogenous PDGF. Interpreting this effect of TNIIIA2, TNIIIA2 induces upregulated expression of PDGF, which in turn stimulates tenascin-C expression and liberation of the TNIIIA2 function. Moreover, independently of PDGF-Rβ expression levels, TNIIIA2 also has the ability to promote *in vitro* disseminative migration of GBM cells through the PKCα signaling pathway. These findings suggest that the tenascin-C/TNIIIA2/PDGF axis could be a potential therapeutic target for GBM.

## 2. Results

### 2.1. TNIIIA2 Stimulated GBM Cell Proliferation in an Autocrine/Paracrine Manner through the Induction of PDGF Expression

**Figure 1:**
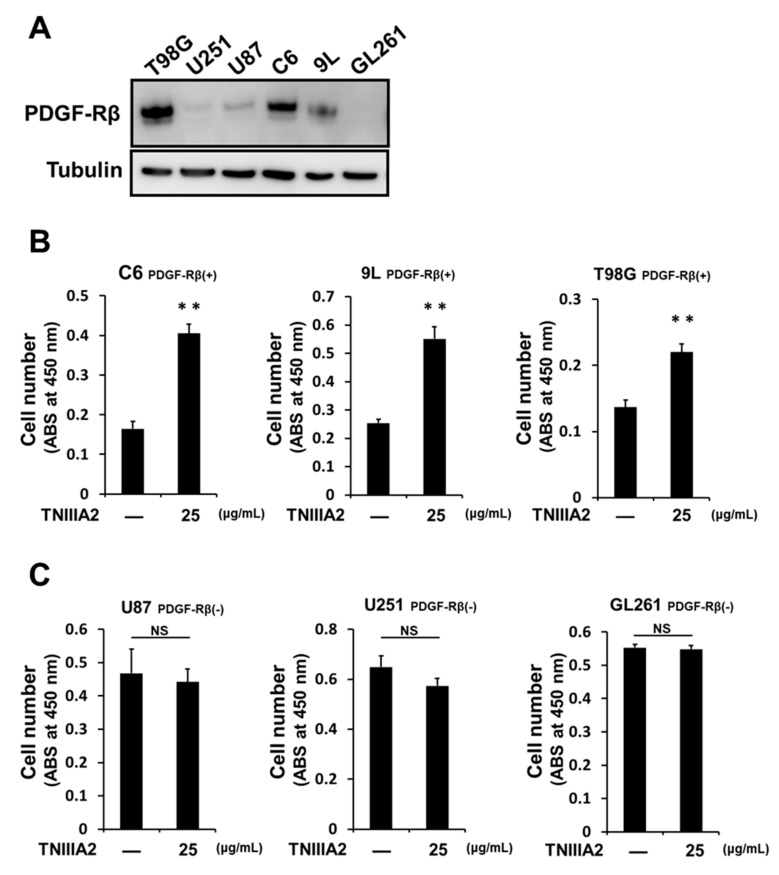
TNIIIA2 promotes the proliferation of glioblastoma multiforme (GBM) cells expressing relatively high quantities of platelet-derived growth factor (PDGF)-Rβ. (**A**) PDGF-Rβ expression in a variety of GBM cell lines assessed by western blotting. (**B**) and (**C**) Effects of TNIIIA2 on proliferation in GBM cells with relatively high (**B**) and low (**C**) levels of PDGF-Rβ expression. GBM cells were treated with TNIIIA2 for 2 days. The number of viable cells was evaluated by WST-8 assay. Data represent the mean ± standard deviation of triplicate determinations, ** *p* < 0.01; NS not significant.

We recently found that activation of integrin α5β1 by TNIIIA2 causes the acquisition of aggressive behavior, such as active proliferation and disseminative migration, in GBM cells. In this study, we further investigated the action mode of the hyper-stimulation of cell proliferation and disseminative migration in GBM cells induced by TNIIIA2. First, we checked the expression of PDGF-Rβ in six GBM cell lines (Figure 1A). Among these cell lines, TNIIIA2 promoted the proliferation of cells expressing relatively high quantities of PDGF-Rβ, such as C6, 9L, and T98G cells, even without the addition of exogenous PDGF (Figure 1B). In contrast, in U87, U251, and GL261 cells, which express low or undetectable levels of PDGF-Rβ, TNIIIA2 hardly promoted proliferation (Figure 1C).

**Figure 2:**
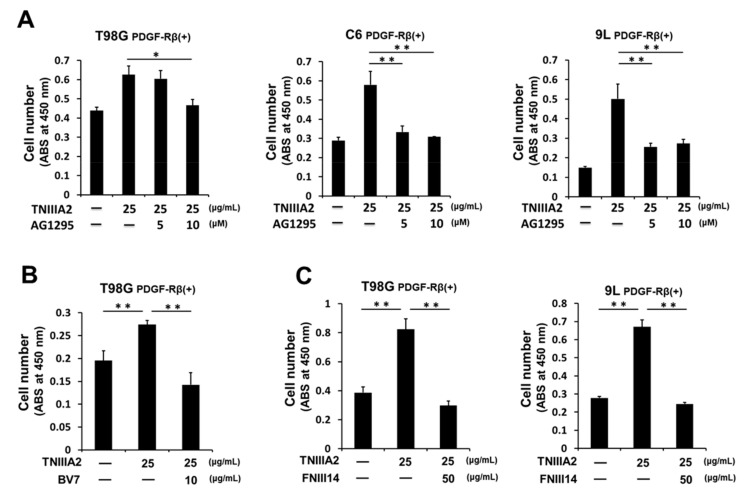
TNIIIA2 potentiates PDGF-dependent GBM cell proliferation through stimulation of PDGF-R tyrosine kinase activity by β1-integrin activation. Effects of (**A**) AG1295, PDGF-R kinase inhibitor, (**B**) BV7, β1-integrin neutralizing mAb, and (**C**) peptide FNIII14, a β1-integrin inactivator on GBM cell proliferation potentiated by TNIIIA2, were examined for cells adhering to the fibronectin substrate, evaluated by WST-8 assay. Data represent the mean ± standard deviation of triplicate determinations, * *p* < 0.05, ** *p* < 0.01.

Interestingly, TNIIIA2-enhanced proliferation was completely overridden by AG1295, a PDGF-R tyrosine kinase inhibitor (Figure 2A), which suggests the role of PDGF-R kinase in TNIIIA2-enhanced proliferation. Additionally, BV7, a function-blocking mAb for β1-integrin, suppressed TNIIIA2-enhanced proliferation, suggesting further association of integrin activation in TNIIIA2-enhanced proliferation (Figure 2B). Supporting these results, peptide FNIII14, which has the specific ability to inactivate β1-integrin [18], abrogated TNIIIA2-enhanced proliferation (Figure 2C).

**Figure 3:**
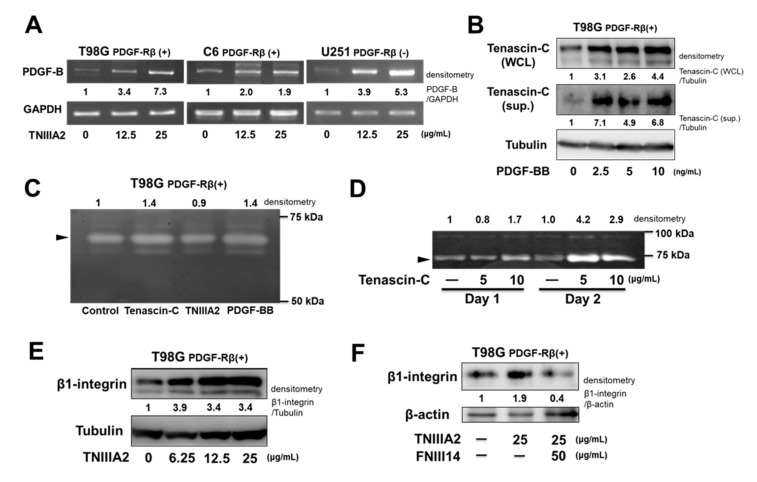
Chain-like increase in PDGF production through the tenascin-C/TNIIIA2/PDGF/MMP-2 positive spiral loop in GBM cells. (**A**) GBM cells were treated with TNIIIA2. After 24 h, PDGF-B mRNA levels were evaluated by semi-quantitative PCR. (**B**) T98G cells were treated with PDGF-BB for 24 h. Cell supernatants (sups.) and whole cell lysates (WCL) were subjected to western blot analysis. (**C**) and (**D**) Gelatinolytic activity of culture supernatants. In panel (**C**), T98G cells were treated with tenascin-C (10 μg/mL), TNIIIA2 (25 μg/mL), or PDGF-BB (10 ng/mL) for 24 h. In panel (**D**), juvenile rat brain-derived cells were treated with tenascin-C at the indicated concentrations. (**E**) and (**F**) Western blotting analysis of the effect of TNIIIA2 on β1-integrin expression in T98G cells. Cells were treated with TNIIIA2 in the absence or presence of peptide FNIII14 for 24 h. Cell lysates were subjected to western blot analysis.

We previously found that integrin α5β1 activation by TNIIIA2 highly promoted PDGF-dependent activation of the PDGF-R kinase; thus, these observations raised the possibility that TNIIIA2-enhanced proliferation without the addition of exogenous PDGF was caused by autocrine/paracrine production of PDGF in TNIIIA2-stimulated cells. This possibility was verified by the result that showed TNIIIA2 induced the upregulation of PDGF-B mRNA expression in T98G and C6 cells (Figure 3A). Treatment of peptide TNIIIA2 also induced upregulation of PDGF-B mRNA levels in U251 cells. No change in cell number was observed upon exposure to peptide TNIIIA2 (please see Figure 1C), probably because of undetectable levels of PDGF-Rβ in U251 cells (please see Figure 1A). Interestingly, PDGF-BB was found to be capable of upregulating the expression of tenascin-C, which is a parental protein molecule of TNIIIA2 (Figure 3B). Pro-adhesive site TNIIIA2 in the tenascin-C molecule could be released upon proteolytic cleavage by inflammatory proteinases, including matrix metalloproteinase (MMP)-2 [11,19]. Therefore, to ascertain whether the expression of MMP could be positively regulated via stimulation with tenascin-C or PDGF, T98G cells were cultured in the presence or absence of tenascin-C, TNIIIA2, and PDGF-BB, and the culture supernatants were subjected to gelatin zymography. As shown in Figure 3C, gelatinolytic activity detected at around 72 kDa, which corresponds to the molecular weight of the active form of MMP-2, was increased by stimulation with either tenascin-C or PDGF-BB in T98G cells. Furthermore, to determine whether cells of GBM tumors produce MMP, brain fibroblasts were isolated from juvenile Wistar rats and examined for MMP activity. Tenascin-C stimulation augmented the gelatinolytic activity detected at 72 kDa, probably due to MMP-2, in rat brain fibroblasts (Figure 3D). Furthermore, TNIIIA2 was also able to upregulate β1-integrin expression (Figure 3E), suggesting that this effect of TNIIIA2 would further increase the stimulation of PDGF-dependent GBM cell proliferation based on β1-integrin activation. Peptide FNIII14 also abrogated this TNIIIA2-dependent upregulation of β1-integrin expression (Figure 3F), as well as the TNIIIA2-enhanced GBM cell proliferation (see Figure 2C). Taken together, TNIIIA2 is able to stimulate PDGF production and subsequently PDGF upregulates the expression of tenascin-C protein in GBM cells. In addition, tenascin-C induced the increased expression of the active form of MMP-2 in GBM cells and brain-derived cells, which might contribute to the continuous production of PDGF, thus suggesting that a positive spiral loop of tenascin-C/TNIIIA2/PDGF signaling leads to hyper-proliferation of GBM cells.

### 2.2. TNIIIA2 Enhanced GBM Migration via β1-Integrin/PKCα Pathway

**Figure 4:**
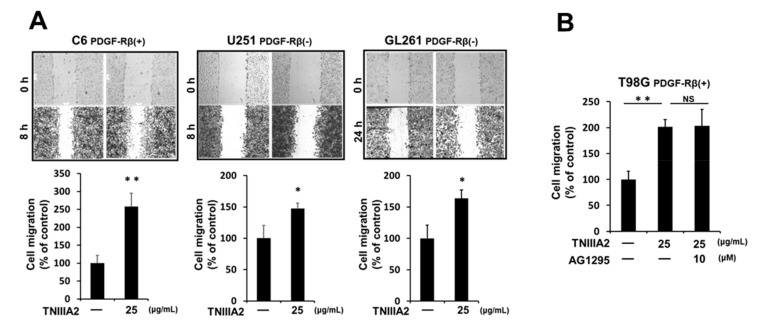
TNIIIA2 promotes cell migration of GBM cells in a PDGF-R-independent manner. (**A**) Effect of TNIIIA2 on cell migration on the fibronectin substrate were evaluated in three GBM cell lines, namely, C6, U251, and GL261, by the wound healing assay, as described in the Materials and Methods section. (**B**) Effect of AG1295, a PDGF-R kinase inhibitor, on TNIIIA2-enhanced T98G cell migration was examined as above. Data represent the mean ± standard deviation of triplicate determinations, * *p* < 0.05, ** *p* < 0.01; NS not significant.

In addition to dysregulated proliferation, GBM is characterized by dissemination throughout the brain parenchyma. Therefore, we next investigated the effect of TNIIIA2 on GBM cell motility via the wound healing assay. TNIIIA2 accelerated GBM cell migration on the fibronectin substrate, which occurred independently of PDGF-Rβ levels (Figure 4A). Potentiated migration by TNIIIA2 was not influenced by AG1295, a PDGF-R tyrosine kinase inhibitor (Figure 4B), suggesting that the molecular mechanism of TNIIIA2-enhanced GBM cell migration was different from that of TNIIIA2-induced hyper-proliferation in GBM cells.

**Figure 5:**
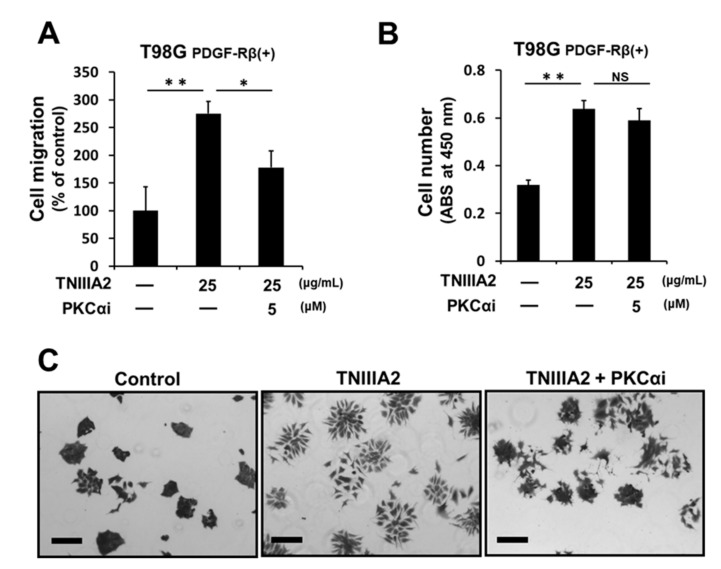
Potentiation of GBM cell scattering and migration by TNIIIA2 is dependent on PKCα activity. T98G cells were treated with TNIIIA2 in the absence or presence of PKCα inhibitor Go6976. (**A**) After 6 h, the migration rate was judged by a wound healing assay. (**B**) After 2 days, the number of viable cells was evaluated by WST-8 assay. Data represent the mean ± standard deviation of triplicate determinations, * *p* < 0.05, ** *p* < 0.01; NS not significant. (**C**) Scattering assay was performed as described in the Materials and Methods section. T98G cells were treated with or without Go6976 (5 μM) for 1 h, and then cultured in the absence or presence of TNIIIA2 (25 μg/mL) for 4 h. Scale bar, 200 μm.

We previously found that β1-integrin activation is induced by binding of TNIIIA2 with syndecan-4 [11]. Since stimulation of syndecan-4 is known to lead to activate downstream signaling necessary for cell migration via PKCα activation [20], we then examined the effect of a PKCα inhibitor on the enhanced proliferation and migration induced by TNIIIA2. PKCα inhibitor Go6976 inhibited TNIIIA2-enhanced migration (Figure 5A) but did not affect TNIIIA2-enhanced proliferation (Figure 5B). We recently found that T98G cells cultured on fibronectin substrate in 2D cultures adhere to each other to form cobblestone-like cell sheets, and TNIIIA2 disrupts the cell-to-cell adhesive interactions, resulting in mesenchymal morphology [13]. As shown in Figure 5C, scattering of T98G cells induced by TNIIIA2 was inhibited by Go6976. Taken together, TNIIIA2-enhanced scattering and migration of GBM cells was controlled by the PKCα-dependent pathway.

## 3. Discussion

**Figure 6:**
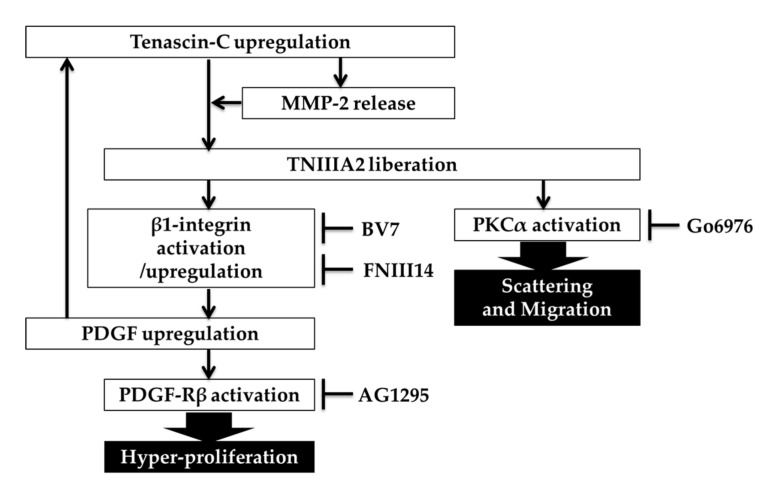
A proposed molecular process of the tenascin-C/TNIIIA2/PDGF positive spiral loop in GBM cell proliferation and the TNIIIA2-induced GBM cell migration and scattering. Tenascin-C produced by GBM cells is cleaved by MMP-2 released by tenascin-C-stimulated GBM cells or fibroblasts. TNIIIA2 liberated from tenascin-C activates β1-integrin and induces upregulation of PDGF, which in turn stimulates the expression of tenascin-C, suggesting a positive feedback loop for hyper-stimulation in GBM cells. TNIIIA2 also induces GBM cell scattering and migration via the PKCα signaling.

Despite high expression of tenascin-C in malignant tumors and a correlation with poor prognosis, the main role of tenascin-C in the pathogenesis of cancer remains unclear. The present study showed that TNIIIA2, which is derived from the cancer-associated variants of tenascin-C, stimulated GBM cell proliferation in an autocrine/paracrine manner through the induction of PDGF expression. Autocrine production of growth factors to secure tumor growth is an important approach employed by tumors for their aggressive progression. PDGF induced by TNIIIA2 was also shown to upregulate the expression of tenascin-C, a parental protein of TNIIIA2, which in turn induced an increase in MMP-2 activity in GBM cells and rat brain-derived fibroblasts, leading to a further liberation of the TNIIIA2 function from tenascin-C molecule. Thus, our findings seem to provide an explanation for the substantial role of highly expressed tenascin-C in oncogenic transformation and aggressive progression in GBM. A number of previous studies support our findings. PDGF-Rβ was reported to be highly expressed by GBM stem cells [14,15]. Some GBM subgroups have high levels of PDGF-B/phosphor-PDGF-Rβ [16,17]: Brennan et al. reported that GBMs can be divided into three subgroups by proteomic analysis of GBM samples, and one of these groups was characterized by high levels of PDGF-B and phosphorylation of PDGF-Rβ [16]. In addition, autocrine and paracrine loops of PDGF signaling are functional for maintaining the growth of glioma cells [21]. GBM highly expresses the cancer-associated tenascin-C variants containing the type III-A2 domain [22], which have the functional site of TNIIIA2. Furthermore, inflammatory proteinases such as MMPs and cathepsins are frequently overexpressed in the tumor microenvironment [23,24,25] and these proteinases can cleave ECM molecules to liberate functional sites concealed within their molecular structures [26]. Taken together, it could be assumed that tenascin-C plays a crucial role in the hyper-stimulation of GBM cell proliferation through the effect of TNIIIA2, where a chain-like increase in PDGF production may occur as follows: inflammation-dependent high expression of tenascin-C, increase in tenascin-C-induced MMP-2, MMP-2-mediated TNIIIA2 liberation, TNIIIA2-induced PDGF expression, and further PDGF-induced tenascin-C expression (Figure 6). The present study also demonstrated that peptide FNIII14 had the ability to break the positive spiral loop of PDGF expression by inhibiting the TNIIIA2 effect, based on the inactivation of β1-integrins. Integrin α5β1 expression is correlated with poor prognosis in GBM [13]. Therefore, this integrin might be considered as a therapeutic target in GBM through its involvement in dysregulated proliferation, disseminative migration, and chemoresistance in GBM cells [27,28,29,30]. We recently reported that peptide FNIII14, which inactivated β1-integrin, can delay GBM tumor growth in a xenograft GBM mouse model [13], suggesting that the tenascin-C/PDGF positive spiral loop might function in vivo. Hence, the anti-tumor effect of peptide FNIII14 observed in our previous study [31,32] might also be interpreted as inhibition of the autocrine production of PDGF in the manner of a spiral loop. In this study, we failed to clear how much PDGF is produced by TNIIIA2 stimulation and to show that it actually functioned to promote cell growth. More detailed analysis, such as determination of the PDGF amount in the culture supernatant by ELISA, will be required.

In addition to active proliferation, GBM is also characterized by dissemination throughout the brain parenchyma. TNIIIA2 enhanced the disseminative migration of GBM cells in a PDGF/PDGF-R-independent manner in an in vitro experiment. We previously reported that TNIIIA2 induces the activation of β1-integrin through a lateral association with syndecan-4 [11]. Syndecan-4 binds to the heparin-binding domain of fibronectin and induces integrin α5β1-mediated cell adhesion [20,33]. Moreover, syndecan-4 activation leads to activation of PKCα and subsequently activates downstream signaling, such as Rho-family GTPases and focal adhesion kinase [20]. Here, we suggested that TNIIIA2-enhanced migration of GBM cells might be partially controlled by PKCα. This notion would be similar to that suggested in a recent study showing that the activation of PKCα promotes GBM cell migration [34,35]. The results of our previous study indicated that TNIIIA2 enhances GBM cell migration through the activation of integrin α5β1 [13]. Taken together, TNIIIA2-enhanced migration might be regulated by β1-integrin/syndecan-4/PKCα pathway. Thus, a strategy using an anti-TNIIIA2 antibody for the treatment of GBM might not be limited among the subgroups of GBM that activates the PDGF signaling pathway. Further investigations are needed to verify the molecular mechanism of TNIIIA2-induced scattering using a spheroid invasion assay or GBM orthotopic model.

In conclusion, this study showed the involvement of tenascin-C/TNIIIA2 in the active growth and migration that characterizes GBM cells. However, to clarify that our findings can be recapitulated in more complex systems close to the living body, it is necessary to further investigate the effect of peptide TNIIIA2 on stem-like phenotypes in glioma stem cells or brain tumor-initiating cells in the future.

## 4. Materials and Methods

### 4.1. Reagents

Human plasma fibronectin was purified as described previously [36]. Tenascin-C was purified as described previously [37]. Peptide TNIIIA2 and FNIII14 has been described previously [11,18]. PDGF-BB was purchased from Wako Pure Chemicals (Tokyo, Japan). PDGF-R kinase inhibitor AG1295 and Go6976, a specific inhibitor of PKC alpha isoforms, were obtained from calbiochem (San Diego, CA, USA). Antibody against PDGF-Rβ (2B3) was purchased from Cell Signaling Technology (Danvers, MA, USA). α-tubulin antibody (DM1A) and β1-integrin (N-20) antibody were purchased from Santa Cruz (Santa Cruz, CA, USA). Tenascin-C antibody (4F10TT) was purchased from IBL (Gunma, Japan). β1-integrin-neutralizing antibody (BV7) was purchased from abcam (Cambridge, UK).

### 4.2. Cell Culture

Human GBM cell line T98G cells, which were obtained from American Type Culture Collection (ATCC, Manassas, VA, USA) were maintained in RPMI1640 medium (Nissui Pharmaceutical, Tokyo, Japan) supplemented with 10% FBS (SAFC Biosciences, Saint Louis, MO, USA). Rat gliosarcoma cell line 9L cells, which were kindly provided by Dr. Yoshida Fumiyo (Department of Neurosurgery, Institute of Clinical Medicine, University of Tsukuba, Japan), rat glioma cell lines C6 cells (ATCC), and human GBM cell U251 cells (ATCC) were maintained in DMEM (Nissui Pharmaceutical) with 10% FBS. Mouse glioma cell line GL261 cells, which were a gift from Dr. Yoshida Fumiyo, were maintained in EMEM (Nissui Pharmaceutical) with 10% FBS. U87 cells (ATCC) were maintained in DMEM containing 4.5 g/L glucose with 10% FBS. These cell lines were passaged in our laboratory soon after receipt from cell banks, and stocked in liquid nitrogen vessels. Each experiment was carried out using thawed cells without further authentication. These cell lines were also authenticated by routine monitoring of cell morphology and proliferation. These cells were cultured up to 15 passages.

### 4.3. Cell Proliferation

T98G, C6, U251, U87, and GL261 (4.0 × 10^3^ cells/well) or 9L (1.0 × 10^4^ cells/well) were seeded on 96-well culture plates coated with fibronectin (0.25 μg/mL) in serum free medium. After culturing for 2 days, the number of viable cells was evaluated by WST-8 assay, as described previously [12].

### 4.4. Western Blotting

T98G cells (2.5 × 10^5^ cells/well) were allowed to adhere in 6-well culture plates coated with fibronectin as above in medium containing 10% FBS and then cells were starved overnight in serum-free medium. Cell collection and subsequent steps were conducted as described previously [12]. Band intensities were quantitated using ImageJ densitometry software (Version 1.51k, National Institutes of Health, Bethesda, MD, USA).

### 4.5. Wound Healing Assay

Cells (2.1 × 10^5^ cells/well) in medium containing 10% FBS were seeded onto 12-well plates coated with fibronectin. A single scratch wound was created using a p200 pipette tip in confluent cells. Cells were washed twice with serum-free media to remove cell debris, supplemented with assay medium. Cells were then fixed with 4% paraformaldehyde and stained with crystal violet, and images were captured by phase-contrast microscopy and analyzed by Motic Image Plus 2.2S (Shimadzu Rika, Kyoto, Japan).

### 4.6. Semiquantitative PCR

The cell RNA was extracted using the PCR RNeasy® Mini Kit (QIAGEN, Hilden, Germany) according to the manufacturer’s instructions. After the RNA concentration was measured by NanoDrop (Thermo Scientific, Waltham, MA, USA), cDNA was gathered by the reverse transcription reaction using Quanti Tect® Reverse Transcription (QIAGEN) and amplified in TaKaRa PCR Thermal Cycler Dicer (Takara Bio, Shiga, Japan) using various primers. The PCR products were electrophoresed using 2% agarose gel TBE including 0.5 µg/mL EtBr and developed with a trans-illuminator. The expression of PDGF and GAPDH were examined using the following primers: PDGF-B primers forward 5′-GATCCGCTCCTTTGATGATC-3’ and reverse 5’-GTCTCACACTTGCATGCCAG-3’; GAPDH primers forward 5’-TTCACCACCATGGAGAAGGC-3’ and reverse 5’-GGCATGGACTGTGGTCATGA-3’. Primers were obtained from Eurofins Genomics (Tokyo, Japan). Band intensities were quantitated using ImageJ densitometry software (Version 1.51k, National Institutes of Health, Bethesda, MD, USA).

### 4.7. Gelatin Zymography

T98G cells (5.0 × 10^4^ cells/well) and rat brain-derived fibroblasts (5.0 × 10^4^ cells/well) in medium containing 10% FBS were seeded onto 48-well plates coated with fibronectin, and then cells were starved overnight in serum-free medium. Cells were washed twice with serum-free media and supplemented with assay medium. Culture supernatants were subjected to the gelatin zymography, as described earlier [38]. Gelatinolytic bands were quantitated using ImageJ densitometry software.

### 4.8. Isolation of Rat Brain-Derived Fibroblasts

Animal procedures were approved (protocol # Y17055; date of approval is 1 April 2017) by the Institutional Animal Care and Use Committee (IACUC) of Tokyo University of Science. Brains were dissected from 3-week old male Wistar rats (Sankyo Laboratory Service, Tokyo, Japan). Dissected brain was minced with scissors, incubated in 0.25% Trypsin-EDTA at 37 °C for 15 min, and dissociated cells were suspended in DMEM with 10% FBS. Cells collected by centrifugation were cultured in DMEM with 10% FBS for 1 day and the nonadherent cells were removed rigorously by replacing the medium. Cells were spread and grown on the plate, which showed bipolar fibroblastic morphology, and then used for gelatin zymography.

### 4.9. Scattering Assay

T98G cells (1.0 × 10^3^ cells/well) were seeded onto 6-well plates, cultured up to formation of a cobblestone-like cell sheet, and then cultured in the assay medium. Cells were then fixed with 4% paraformaldehyde and stained with crystal violet, and cell images were captured by phase-contrast microscopy.

### 4.10. Statistical Analysis

Data are expressed as the mean ± standard deviation. Two-tailed Student t test or one-way analysis of variance (ANOVA) followed by Student-Newman-Keuls post-hoc test was used to determine statistical differences. Values of *p* < 0.05 were considered significant.

## Abbreviations

PDGF	platelet-derived growth factor
GBM	glioblastoma multiforme
ECM	extracellular matrix
PDGF-R	platelet-derived growth factor receptor
PKC	protein kinase C
RPMI	Roswell Park Memorial Institute
FBS	fetal bovine serum
DMEM	Dulbecco’s Modified Eagle’s Medium
PCR	polymerase chain reaction
TBE	tris-borate-EDTA
EDTA	ethylenediaminetetraacetic acid
EtBr	ethidium bromide
MMP	matrix metalloproteinase
sup.	Supernatant
WCL	whole cell lysates
